# Does Massage Help Athletes After Exercise?

**DOI:** 10.31486/toj.20.0008

**Published:** 2020

**Authors:** Michelle Sriwongtong, Joshua Goldman, Yuka Kobayashi, Andrew W. Gottschalk

**Affiliations:** ^1^ Department of Family Medicine, Division of Sports Medicine, University of California, Los Angeles, Los Angeles, CA; ^2^ Department of Family Medicine, Oregon Health and Sciences University, Portland, OR; ^3^ Department of Orthopedics, Sports Medicine Institute, Ochsner Clinic Foundation, New Orleans, LA

## SPORTS AND MEDICINE AT THE HIGHEST LEVELS OF PLAY

During the 2018 FIFA (Fédération Internationale de Football Association) World Cup, France's national football team—the eventual champions—played 7 soccer matches in 30 days.^1^ Similarly, the Union of European Football Associations teams regularly compete in 9 or more games per month.^2^ Big-name players such as Cristiano Ronaldo and big-name managers such as José Mourinho have discussed the importance of rest between matches and the need for improved muscle recovery methods.^3^ The increasing prevalence of schedules such as these encourages players and team medical staff to find ways to expedite athletes’ recovery.

## SPORTS MEDICINE AND MASSAGE

Massage therapy has existed for millennia and remains popular among athletes and nonathletes alike.^4^ A 2013 review of soccer recovery strategies found that 78% of French professional teams utilized massage therapy to help athletes recover.^5^ A 1992 Canadian review of sports massage by Cafarelli and Flint concluded that up to 45% of total time in physiotherapy for sport-related injury and performance consisted of massage treatments.^6^

Sports massage is not a single modality but a combination of massage techniques such as effleurage (sliding, circular movements), petrissage (tissue kneading and/or pressure), friction (pressure application), and application of vibrations.^4^ The proposed physiologic benefits of massage are myriad. The American Massage Therapy Association alone lists relief of muscle tension and stiffness; faster healing of strained muscles and sprained ligaments; reduced muscle pain, swelling, and spasm; greater joint flexibility and range of motion; and enhanced athletic performance as benefits of massage.^4,7,8^ Other proposed physiologic benefits include alleviation of myofascial pain and spasm, improvement in blood flow, and expedited clearance of both lactate and creatine kinase from the blood.^4,9^

The heterogeneity in massage techniques, paired with this lack of agreement as to what exactly the physical benefits are, makes the scientific study of massage in athletes challenging. The outcomes measured in massage studies are likewise diverse and include joint range of motion, isokinetic and isometric peak strength, vertical and long jumps, and blood lactate and creatine kinase levels.^4^

## REVIEW OF EVIDENCE

A 2008 systematic review by Best et al analyzed studies that assessed muscle recovery and performance after massage following strenuous exercise.^4^ Seventeen studies fitting the inclusion criteria were case series. Ten of the 17 studies evaluated postexercise function only, and only 2 demonstrated improved muscle function after massage. The other 7 case series evaluated postexercise function and delayed onset muscle soreness (DOMS) subjective symptoms. Four of the 7 studies demonstrated massage-associated relief from DOMS symptoms. Somewhat more encouraging, the 10 randomized controlled trials that fit the inclusion criteria for the Best et al review demonstrated what the authors called “moderate evidence” that massage may be effective in muscle recovery. No adverse effects from massage therapy were reported in any of the 27 studies included in the review.^4^

A 2013 review of soccer players’ recovery strategies by Nédélec et al reached conclusions similar to those of Best et al but went a step further by cautioning, “It should not be excluded that massage of injured tissue may lead to further damage in muscle if given immediately after a training session that induced muscle damage.”^5^

A 2016 study by Nunes et al evaluated massage in Ironman athletes. The authors found that a 7-minute postevent massage resulted in statistically significant decreases in athletes’ subjective pain and fatigue scores on a visual analog scale (VAS) scale of 0 to 100 mm (a 100-mm line anchored by 2 verbal descriptors for each extreme end of symptoms) compared to athletes who instead rested in a seated position for 7 minutes. The massage group had lower perceived pain VAS scores by an average of 7 mm (95% confidence interval [CI] 1-13) and lower perceived fatigue scores by an average of 15 mm (95% CI 9-21).^10^

Regarding the ideal duration of massage therapy, a 2016 metaanalysis found that shorter massages (5 to 12 minutes) have superior outcomes compared to longer massages (13+ minutes), with objective improvements in work on a cycling sprint, endurance, and vertical jump. Even so, the analysis concludes, “The effects of massage on performance recovery are rather small…it remains questionable if the limited effects justify the widespread use of massage as a recovery intervention in competitive athletes.”^9^

Massage therapy does seem to provide positive psychological benefits to athletes. In 1988, Weinberg et al concluded that massage significantly decreased levels of tension, confusion, fatigue, anxiety, and depression in university students in physical education classes compared to controls.^11^

More than a decade later in 2000, Hemmings et al studied amateur boxers who received either massage or passive rest after performing on a boxing ergometer. After the interventions, a Wilcoxon matched-pairs test showed that perceptions of recovery were significantly higher (*P*<0.01) in the massage group. When comparing the groups, the same study found no differences in punching force or blood glucose concentrations. Interestingly, the authors did find significant elevations in blood lactate concentrations in the massage group, perhaps adding weight to Nédélec's words of caution noted earlier.^12^

## EVIDENCE-BASED SPORTS MEDICINE

Current literature provides scant evidence supporting the use of massage for physiologic muscle recovery. Evidence does support the use of massage for positive psychological effects.^11^

## SPORTS MEDICINE AND ANECDOTE

Professional European soccer teams face several pivotal matches in the final stages of the Champions League tournament each season. As Poppendieck et al wrote, “…the fact that an athlete ‘feels better’ after receiving a massage might be sufficient to justify its use despite the absence of measurable physiological benefits.”^9^ As such, athletes and their team medical staffs will likely continue to use massage as one intervention to “aid” in muscle recovery despite ongoing lack of conclusive evidence supporting its use for this purpose.

## CONCLUSION

If an intervention makes an athlete feel better after exercise, that is a positive, evidence-based outcome. The danger is when an intervention is sold—many times literally—as having positive microscopic or macroscopic physiologic outcomes when, even after repeat studies, none has been proven. Situations such as these pave the way for not just poor medicine, but dangerous medicine. As medical professionals, our obligation to consider nonmaleficence—or *do no harm—*can certainly be extrapolated to include *do not lie* about the physiologic effects of an intervention. An extreme example is the Cochrane review “Massage for promoting mental and physical health in typically developing infants under the age of six months.” The authors reviewed 34 studies that, as with the adult studies we have discussed here, also collectively failed to demonstrate physiologic benefits of massage, only this time after applying that intervention to babies in their first months of life.^13^

## Notes

This article meets the Accreditation Council for Graduate Medical Education and the American Board of Medical Specialties Maintenance of Certification competencies for Patient Care and Medical Knowledge.

## References

[1] FIFA World Cup Russia 2018 Match Schedule. resources.fifa.com/mm/Document/Tournament/Competition/02/66/71/62/2018fwc_matchschedule_02092016_EN_Neutral.pdf. Published September 2, 2016 Accessed January 5, 2020.

[2] Matches. Union of European Football Associations. www.uefa.com/uefaeuro-2020/matches/#/md/33673/. Accessed January 5, 2020.

[3] Scoppa V. Fatigue and team performance in soccer: evidence from the FIFA World Cup and the UEFA European Championship. J Sports Econom. 2015 Jun 1;16(5):482-507. doi: 10.1177/1527002513502794.

[4] Best TM, Hunter R, Wilcox A, Haq F Effectiveness of sports massage for recovery of skeletal muscle from strenuous exercise. Clin J Sport Med. 2008 9;18(5):446-460. doi: 10.1097/JSM.0b013e31818837a1. 18806553

[5] Nédélec M, McCall A, Carling C, Legall F, Berthoin S, Dupont G Recovery in soccer: part ii-recovery strategies. Sports Med. 2013 1;43(1):9-22. doi: 10.1007/s40279-012-0002-0. 23315753

[6] Cafarelli E, Flint F. The role of massage in preparation for and recovery from exercise. An overview. Sports Med. 1992 7;14(1):1-9. doi: 10.2165/00007256-199214010-00001. 1641539

[7] Massage therapy for those who exercise. American Massage Therapy Association. www.amtamassage.org/about/position-statements/massage-therapy-for-those-who-exercise/. Accessed January 5, 2020.

[8] Kong PW, Chua YH, Kawabata M, Burns SF, Cai C Effect of post-exercise massage on passive muscle stiffness measured using myotonometry – a double-blind study. J Sports Sci Med. 2018 Nov 20;17(4):599-606. 30479528 PMC6243630

[9] Poppendieck W, Wegmann M, Ferrauti A, Kellmann M, Pfeiffer M, Meyer T Massage and performance recovery: a meta-analytical review. Sports Med. 2016 2;46(2):183-204. doi: 10.1007/s40279-015-0420-x. 26744335

[10] Nunes GS, Bender PU, de Menezes FS, Yamashitafuji I, Vargas VZ, Wageck B Massage therapy decreases pain and perceived fatigue after long-distance Ironman triathlon: a randomised trial. J Physiother. 2016 4;62(2):83-87. doi: 10.1016/j.jphys.2016.02.009. 27025688

[11] Weinberg R, Jackson A, Kolodny K The relationship of massage and exercise to mood enhancement. Sport Psychol. 1988;2(3):202-211. doi: 10.1123/tsp.2.3.202.

[12] Hemmings B, Smith M, Graydon J, Dyson R Effects of massage on physiological restoration, perceived recovery, and repeated sports performance. Br J Sports Med. 2000 4;34(2):109-114; discussion 115. doi: 10.1136/bjsm.34.2.109. 10786866 PMC1724183

[13] Bennett C, Underdown A, Barlow J Massage for promoting mental and physical health in typically developing infants under the age of six months. Cochrane Database Syst Rev. 2013 Apr 30;(4):CD005038. doi: 10.1002/14651858.CD005038.pub3. 23633323 PMC8078453

